# Supplementary figures and images for: Resequencing and phenotyping of the first highly inbred eggplant multiparent population reveal SmLBD13 as a key gene associated with root morphology
Source: Hortic Res. 2025 Jun 26;12(9):uhaf167. doi: 10.1093/hr/uhaf167 (PMC12344552; doi:10.1093/hr/uhaf167)

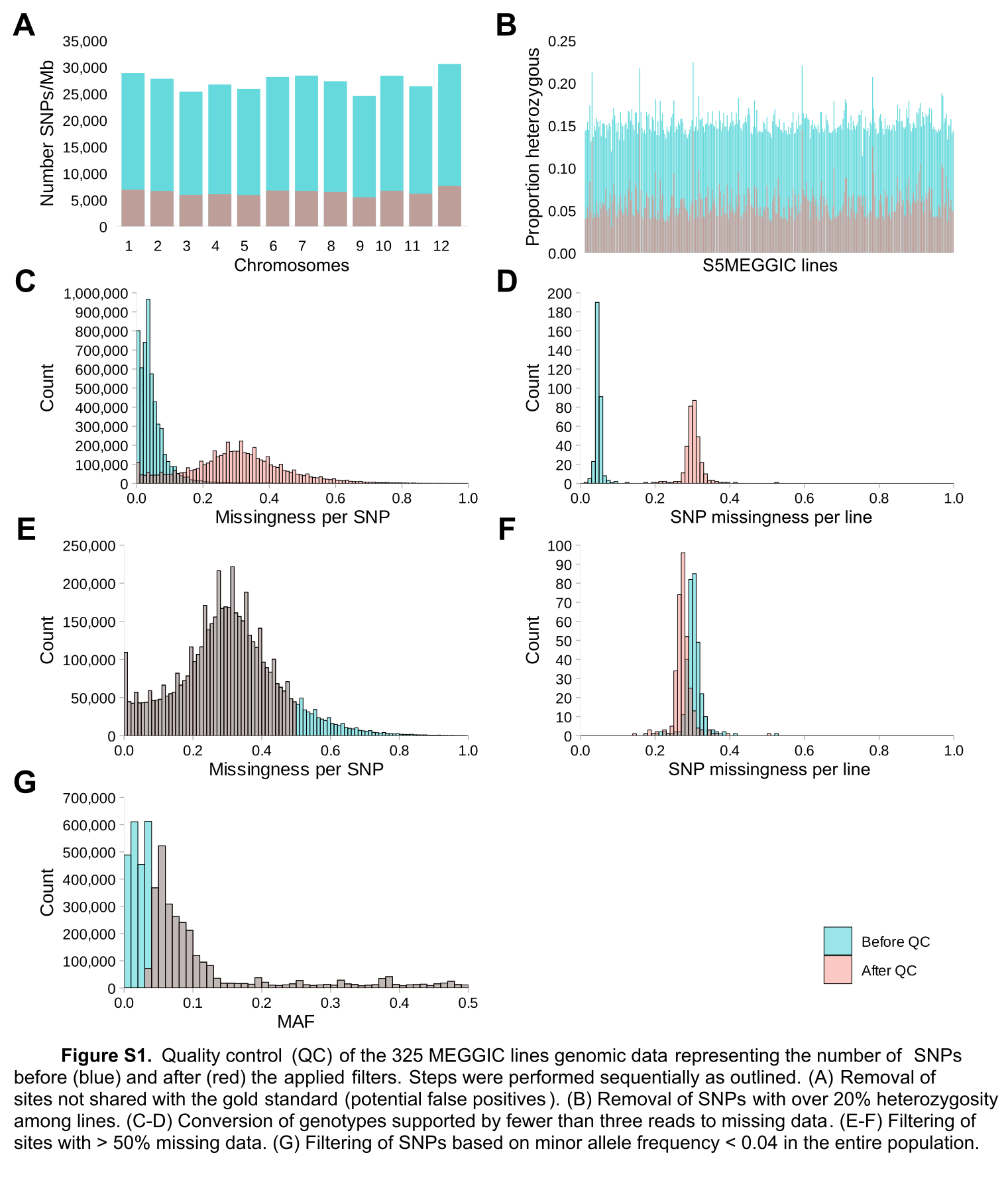

Supplement: Web_Material_uhaf167 [file web_material_uhaf167.zip › Figure_S1.tiff]

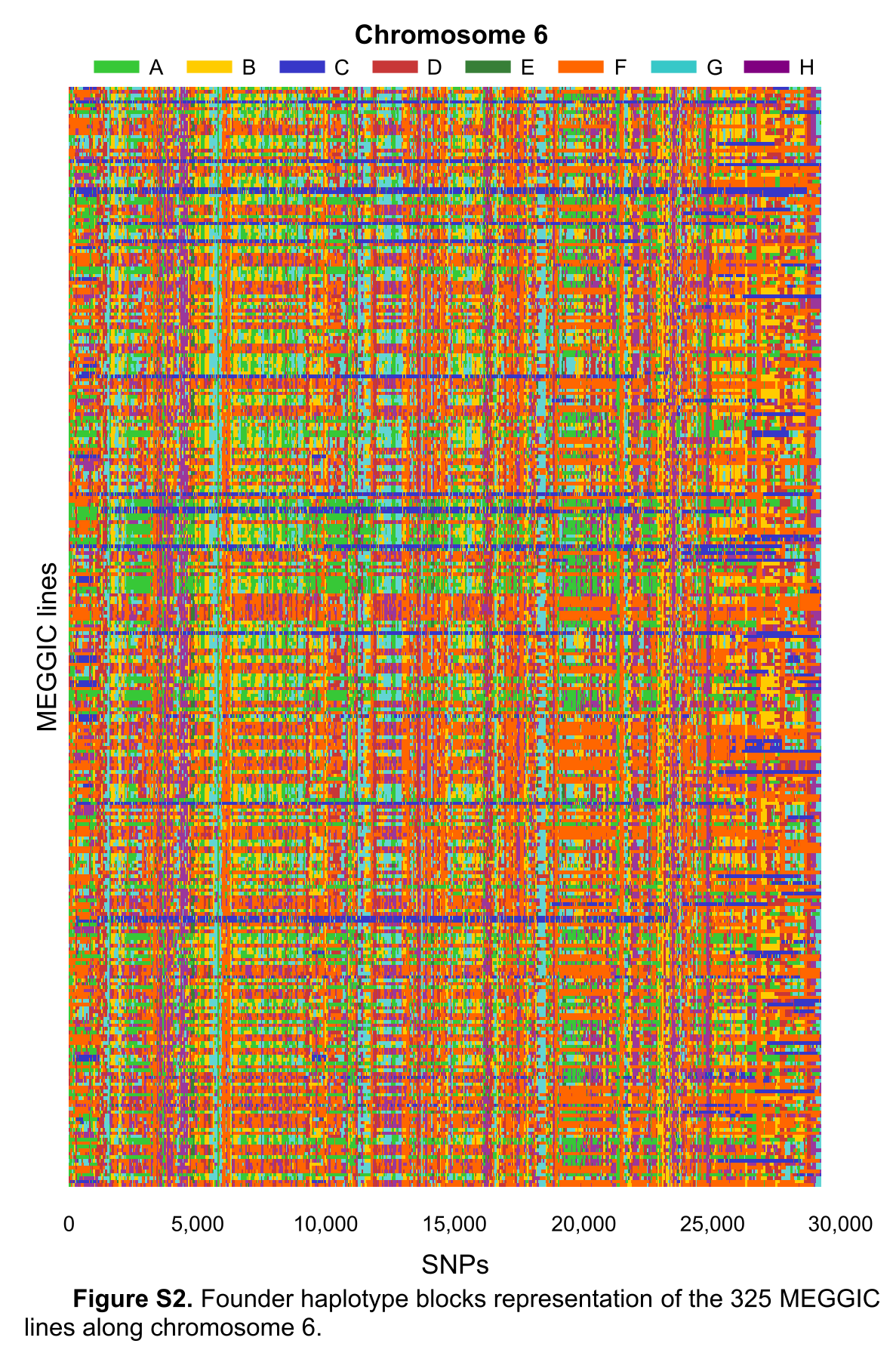

Supplement: Web_Material_uhaf167 [file web_material_uhaf167.zip › Figure_S2.tiff]

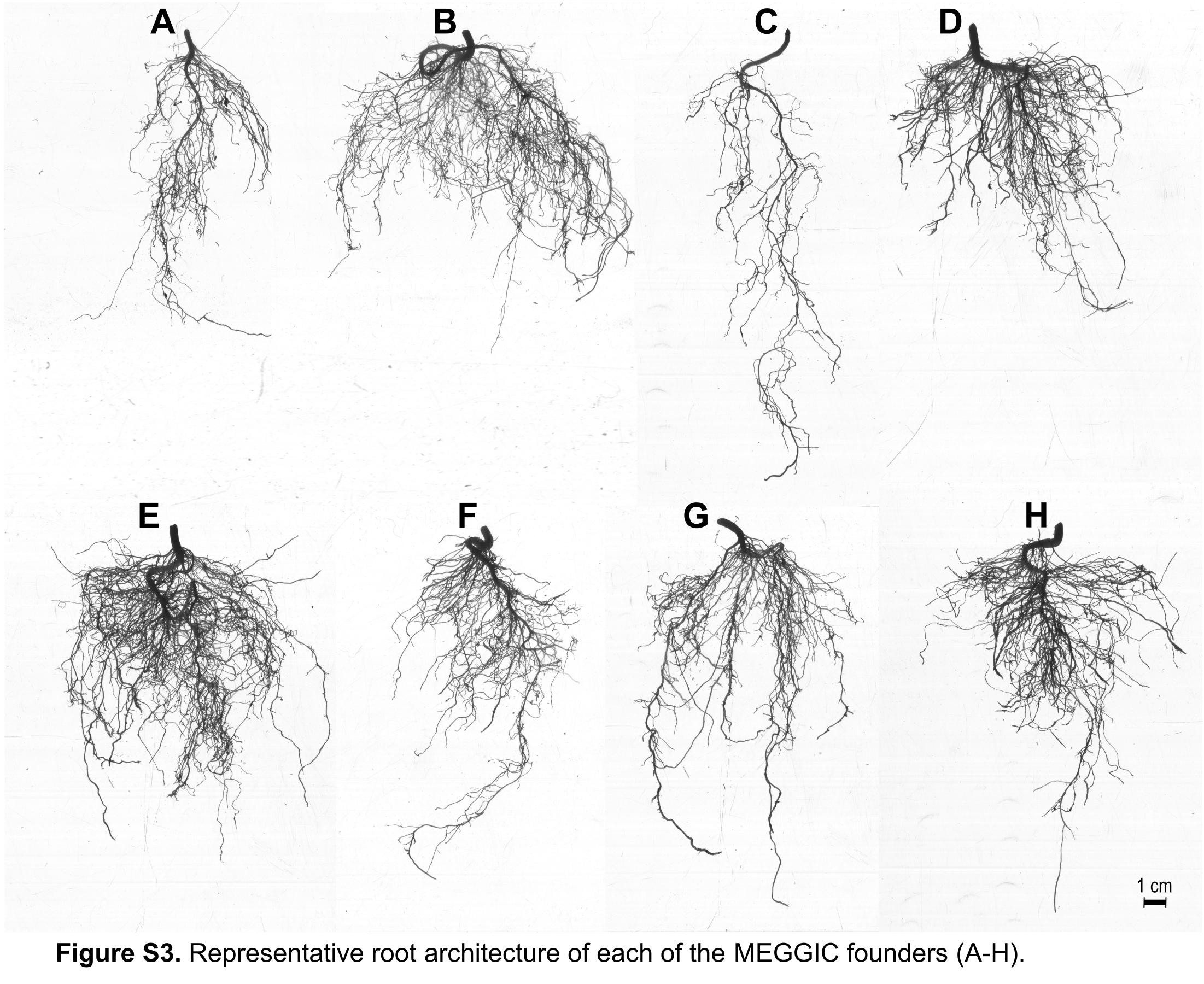

Supplement: Web_Material_uhaf167 [file web_material_uhaf167.zip › Figure_S3.tiff]

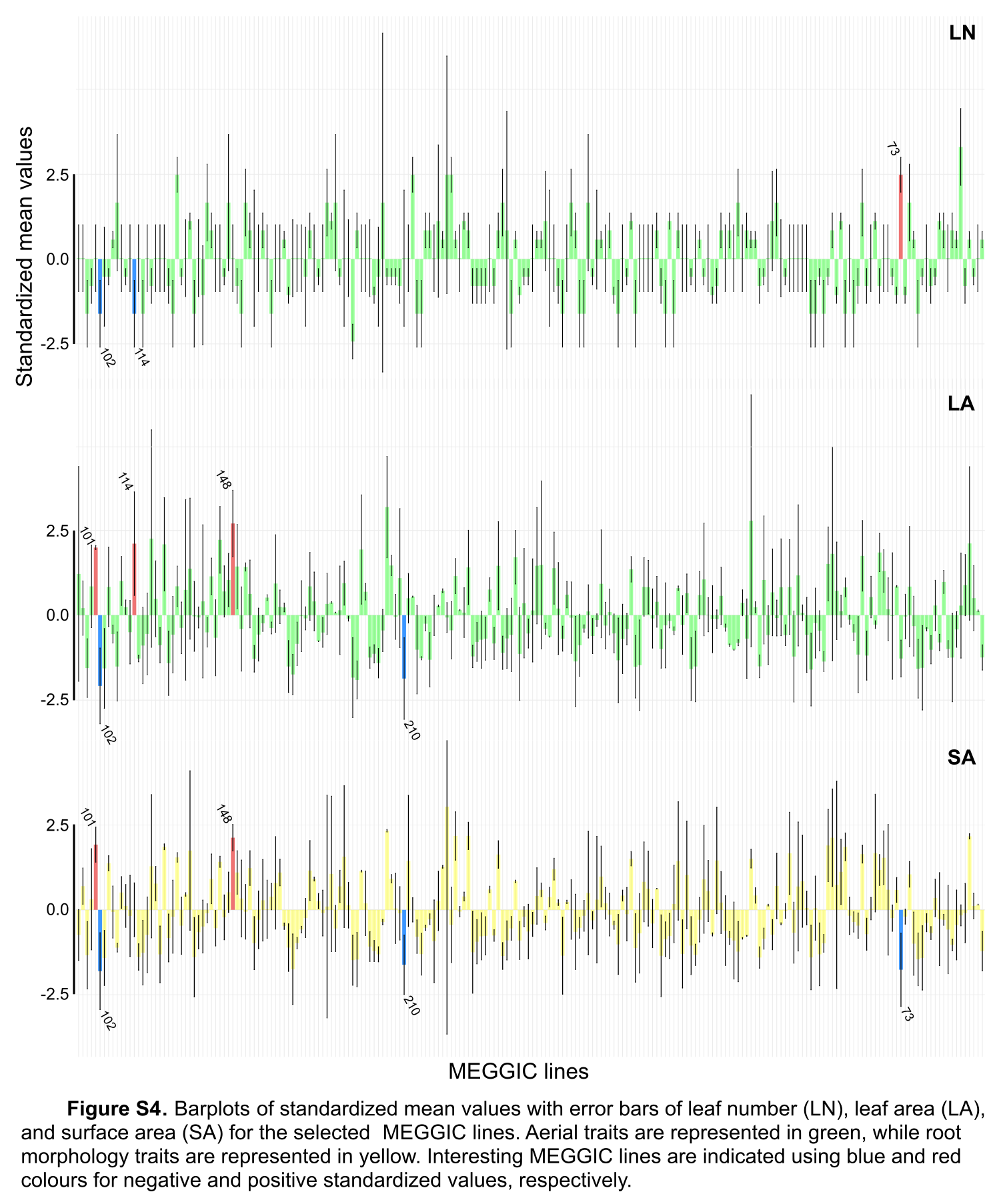

Supplement: Web_Material_uhaf167 [file web_material_uhaf167.zip › Figure_S4.tiff]

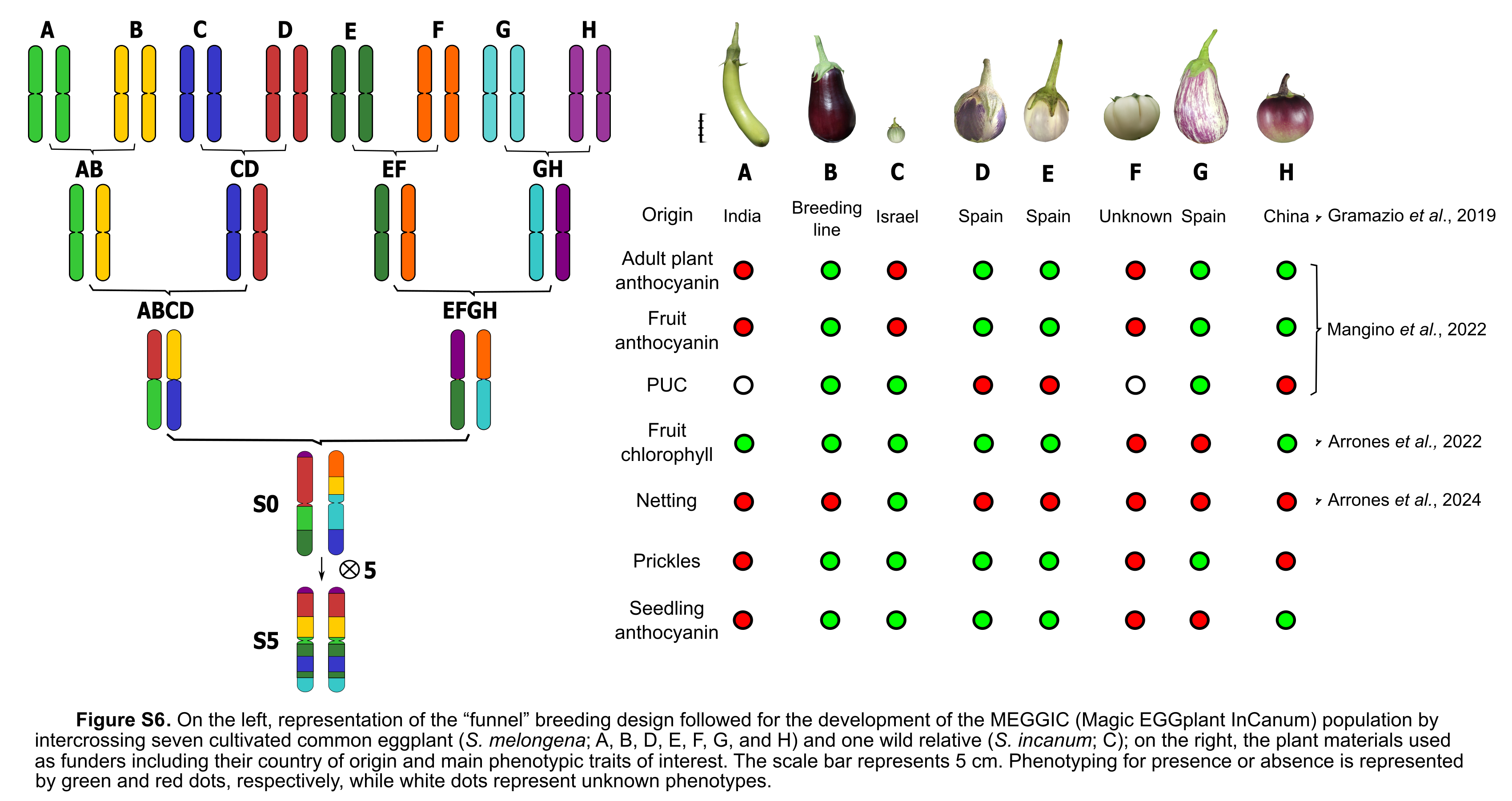

Supplement: Web_Material_uhaf167 [file web_material_uhaf167.zip › Figure_S6.tiff]

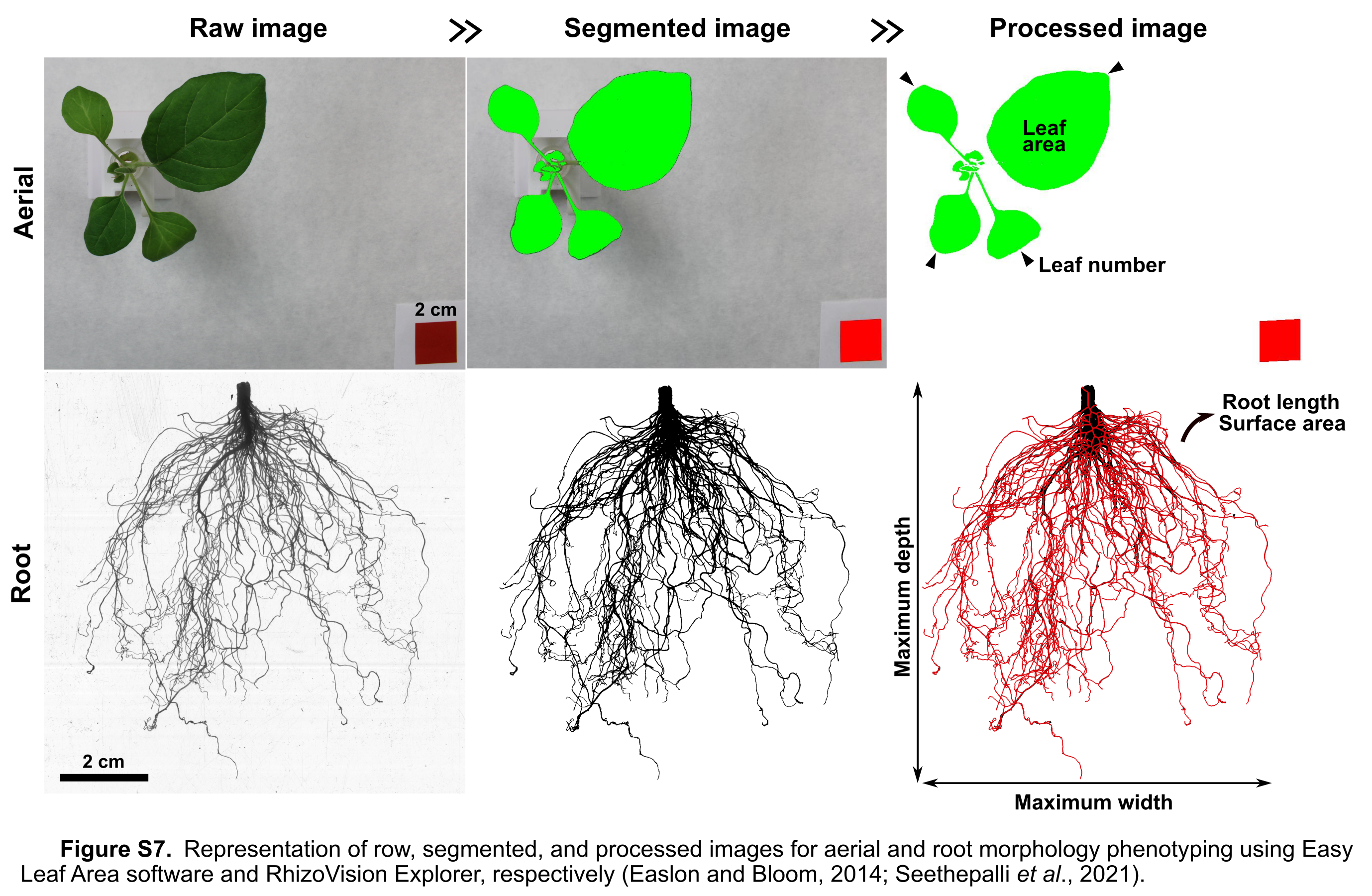

Supplement: Web_Material_uhaf167 [file web_material_uhaf167.zip › Figure_S7.tiff]

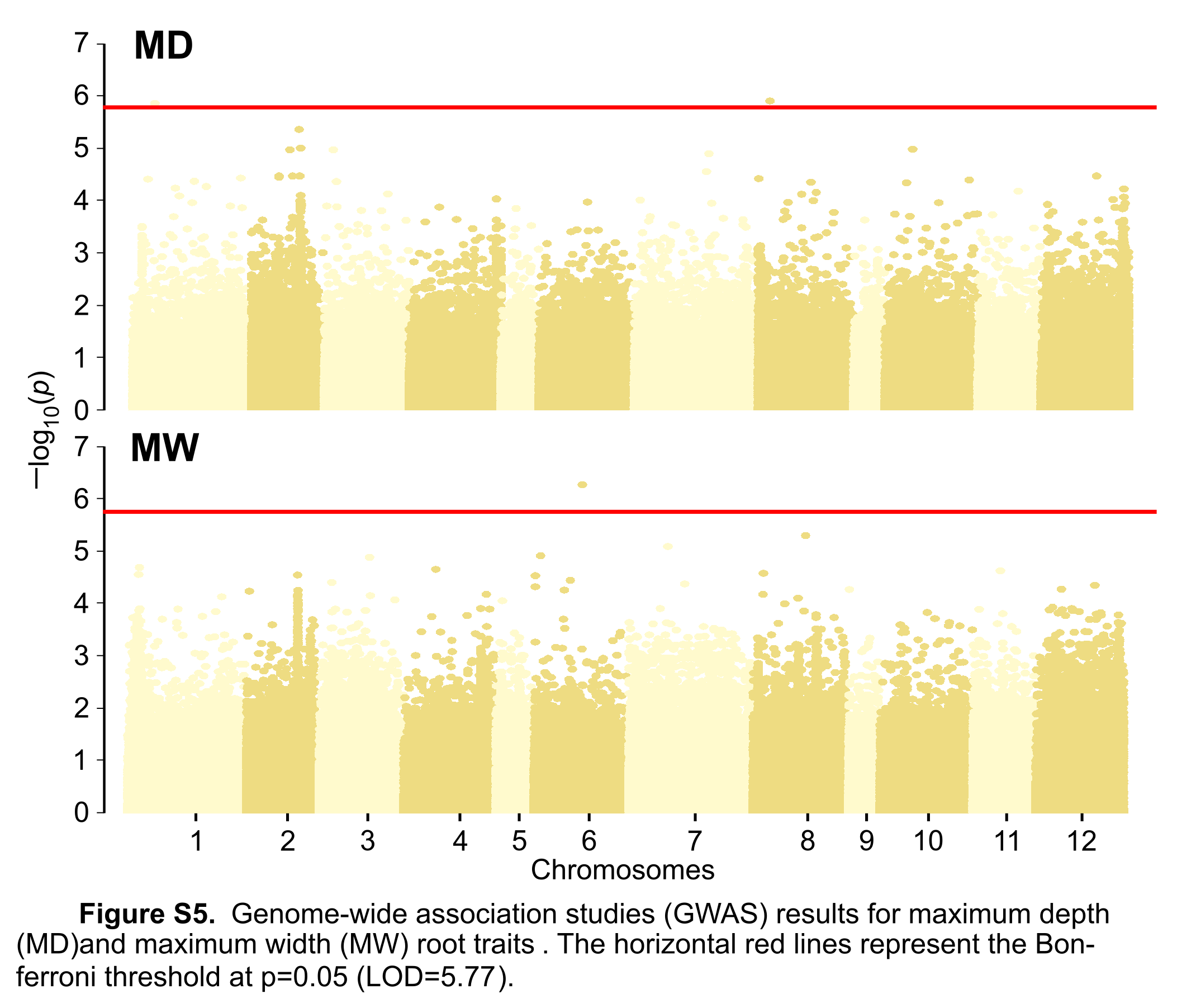

Supplement: Web_Material_uhaf167 [file web_material_uhaf167.zip › Figures_S5.tiff]
